# Crystal structure of ethyl 2-chloro-6-methyl­quinoline-3-carboxyl­ate

**DOI:** 10.1107/S1600536814016900

**Published:** 2014-08-01

**Authors:** Hasna Hayour, Abdelmalek Bouraiou, Sofiane Bouacida, Saida Benzerka, Ali Belfaitah

**Affiliations:** a Laboratoire des Produits Naturels d’Origine Végétale et de Synthèse Organique, PHYSYNOR, Université Constantine 1, 25000 Constantine, Algeria; b Unité de Recherche de Chimie de l’Environnement et Moléculaire Structurale, CHEMS, Université Constantine 1, 25000 , Algeria; c Département Sciences de la Matière, Faculté des Sciences Exactes et Sciences de la Nature et de la Vie, Université Oum El Bouaghi, Algeria

**Keywords:** crystal structure, 2-chloro-3-formyl­quinoline, ethyl ester, π–π stacking

## Abstract

In the title compound, C_13_H_12_ClNO_2_, the dihedral angle between the planes of the quinoline ring system (r.m.s. deviation = 0.029 Å) and the ester group is 54.97 (6)°. The C—O—C—C_m_ (m = meth­yl) torsion angle is −140.62 (16)°. In the crystal, mol­ecules inter­act *via* aromatic π–π stacking [shortest centroid–centroid separation = 3.6774 (9) Å] generating (010) sheets.

## Related literature   

For background to 2-chloro-3-formyl­quinolines, see: Michael (2004 ▶); Abdel-Wahab *et al.* (2012 ▶). For our previous work in this area, see: Benzerka *et al.* (2012 ▶, 2013 ▶).
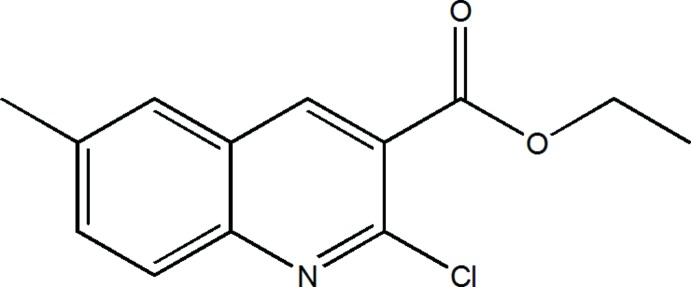


## Experimental   

### Crystal data  

C_13_H_12_ClNO_2_
*M* *_r_* = 249.69
Triclinic, 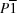

*a* = 6.0391 (5) Å
*b* = 7.2986 (6) Å
*c* = 13.4323 (12) Å
α = 98.238 (6)°
β = 90.123 (5)°
γ = 96.429 (6)°
*V* = 582.16 (9) Å^3^
*Z* = 2
Mo *K*α radiation
μ = 0.32 mm^−1^
*T* = 150 K
0.18 × 0.14 × 0.12 mm

### Data collection  

Bruker APEXII diffractometer
Absorption correction: multi-scan (*SADABS*; Bruker, 2006 ▶) *T*_min_ = 0.690, *T*_max_ = 0.747
5190 measured reflections
2061 independent reflections
1872 reflections with *I* > 2σ(*I*)
*R*_int_ = 0.016

### Refinement  

*R*[*F* ^2^ > 2σ(*F* ^2^)] = 0.028
*wR*(*F* ^2^) = 0.078
*S* = 1.06
2061 reflections
156 parameters
H-atom parameters constrained
Δρ_max_ = 0.25 e Å^−3^
Δρ_min_ = −0.20 e Å^−3^

### 

Data collection: *APEX2* (Bruker, 2006 ▶); cell refinement: *SAINT* (Bruker, 2006 ▶); data reduction: *SAINT*; program(s) used to solve structure: *SIR2002* (Burla *et al.*, 2005 ▶); program(s) used to refine structure: *SHELXL97* (Sheldrick, 2008 ▶); molecular graphics: *ORTEP-3 for Windows* (Farrugia, 2012 ▶) and *DIAMOND* (Brandenburg & Berndt, 2001 ▶); software used to prepare material for publication: *WinGX* (Farrugia, 2012 ▶).

## Supplementary Material

Crystal structure: contains datablock(s) I. DOI: 10.1107/S1600536814016900/hb7259sup1.cif

Structure factors: contains datablock(s) I. DOI: 10.1107/S1600536814016900/hb7259Isup2.hkl

Supporting information file. DOI: 10.1107/S1600536814016900/hb7259Isup3.cml

**Fig. 1.**
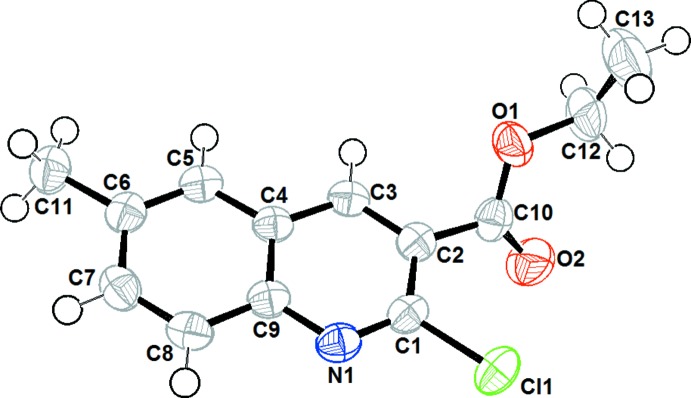
The structure of the title compound with displacement ellipsoids drawn at the 50% probability level.

**Fig. 2.**
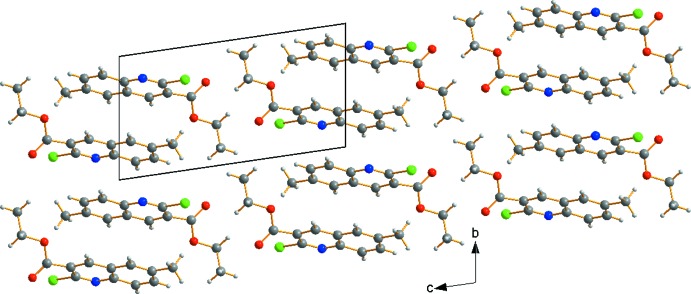
A diagram of the layered crystal packing of (I) viewed down the a axis.

. DOI: 10.1107/S1600536814016900/hb7259fig1.tif
The structure of the title compound with displacement ellipsoids drawn at the 50% probability level.

a . DOI: 10.1107/S1600536814016900/hb7259fig2.tif
A diagram of the layered crystal packing of (I) viewed down the *a* axis.

CCDC reference: 1015360

Additional supporting information:  crystallographic information; 3D view; checkCIF report

## supplementary crystallographic information

### S1. Comment

The 2-chloro-3-formylquinolines occupy a prominent position as key intermediates for further annelation and various functional group inter-conversions (Abdel-Wahab *et al.*, 2012; Michael, 2004). As part of our ongoing studies in this area (Benzerka *et al.*, 2012, 2013), we now describe the synthesis and single-crystal X-ray structure of the title compound, (I).

The molecular geometry and the atom-numbering scheme of (I) are shown in Fig. 1. In the asymmetric unit of title compound the quinoline ring is three times substituted by two methyl, one chlore and one ethyl carboxylate. The crystal packing can be described as double layers parallel to (010) plane (Fig. 2). It features π···π stacking, distances controid-controid between aromatic rings are from 3.6774 (9) to 4.2262 (9) Å.

### S2. Experimental

Into a solution of NaCN (3 mmol) in absolute ethanol (15 ml), was added, in portion and at 0 °C,a mixture of 1 mmol of 2-chloro-3-formyl-6-methylquinoline and activated manganese dioxide (6.7 mmol). The reaction mixture was stirred for 3 h at rt. Purification of the corresponding compound was carried out by diluting the reaction mixture with CH_2_Cl_2_ and filtering through a small column packed with 4 cm of celite and 3 cm of silica gel. The pure compound was recovered after evaporation of solvents. Colourless blocks of (I) were obtained by dissolving the pure compound in EtOH and allowing the solution to slowly evaporate at room temperature.

### S3. Refinement

All H atoms were localized on Fourier maps but introduced in calculated positions and treated as riding on their parent C atom. (with C—H = 0.93 (aromatic), 0.96 (methyl) and 0.97 Å (methylene) and *U*_iso_(H) =1.5 or 1.2(carrier atom).

### Crystal data

**Table:**

C_13_H_12_ClNO_2_	*Z* = 2
*M**_r_* = 249.69	*F*(000) = 260
Triclinic, *P*1	*D*_x_ = 1.424 Mg m^−^^3^
*a* = 6.0391 (5) Å	Mo *K*α radiation, λ = 0.71073 Å
*b* = 7.2986 (6) Å	Cell parameters from 2875 reflections
*c* = 13.4323 (12) Å	θ = 2.8–25.0°
α = 98.238 (6)°	µ = 0.32 mm^−^^1^
β = 90.123 (5)°	*T* = 150 K
γ = 96.429 (6)°	BLOCK, colourless
*V* = 582.16 (9) Å^3^	0.18 × 0.14 × 0.12 mm

### Data collection

**Table:**

Bruker APEXII diffractometer	1872 reflections with *I* > 2σ(*I*)
Graphite monochromator	*R*_int_ = 0.016
CCD rotation images, thin slices scans	θ_max_ = 25.1°, θ_min_ = 2.8°
Absorption correction: multi-scan (*SADABS*; Bruker, 2006)	*h* = −7→7
*T*_min_ = 0.690, *T*_max_ = 0.747	*k* = −8→8
5190 measured reflections	*l* = −16→15
2061 independent reflections	

### Refinement

**Table:**

Refinement on *F*^2^	Primary atom site location: structure-invariant direct methods
Least-squares matrix: full	Secondary atom site location: difference Fourier map
*R*[*F*^2^ > 2σ(*F*^2^)] = 0.028	Hydrogen site location: inferred from neighbouring sites
*wR*(*F*^2^) = 0.078	H-atom parameters constrained
*S* = 1.06	*w* = 1/[σ^2^(*F*_o_^2^) + (0.0383*P*)^2^ + 0.2333*P*] where *P* = (*F*_o_^2^ + 2*F*_c_^2^)/3
2061 reflections	(Δ/σ)_max_ < 0.001
156 parameters	Δρ_max_ = 0.25 e Å^−^^3^
0 restraints	Δρ_min_ = −0.20 e Å^−^^3^

### Special details

**Table:**

Geometry. All e.s.d.'s (except the e.s.d. in the dihedral angle between two l.s. planes) are estimated using the full covariance matrix. The cell e.s.d.'s are taken into account individually in the estimation of e.s.d.'s in distances, angles and torsion angles; correlations between e.s.d.'s in cell parameters are only used when they are defined by crystal symmetry. An approximate (isotropic) treatment of cell e.s.d.'s is used for estimating e.s.d.'s involving l.s. planes.
Refinement. Refinement of *F*^2^ against ALL reflections. The weighted *R*-factor *wR* and goodness of fit *S* are based on *F*^2^, conventional *R*-factors *R* are based on *F*, with *F* set to zero for negative *F*^2^. The threshold expression of *F*^2^ > σ(*F*^2^) is used only for calculating *R*-factors(gt) *etc*. and is not relevant to the choice of reflections for refinement. *R*-factors based on *F*^2^ are statistically about twice as large as those based on *F*, and *R*- factors based on ALL data will be even larger.

### Fractional atomic coordinates and isotropic or equivalent isotropic displacement parameters (Å^2^)

**Table:**

	*x*	*y*	*z*	*U*_iso_*/*U*_eq_	
C10	0.6109 (2)	0.4100 (2)	0.31856 (11)	0.0233 (3)	
C11	0.7716 (3)	0.2270 (2)	−0.23686 (11)	0.0281 (4)	
H11A	0.7218	0.3141	−0.2768	0.042*	
H11B	0.7582	0.105	−0.2757	0.042*	
H11C	0.9246	0.2644	−0.2169	0.042*	
C12	0.7931 (3)	0.6667 (2)	0.42998 (12)	0.0356 (4)	
H12A	0.9531	0.6636	0.4327	0.043*	
H12B	0.7258	0.5967	0.4805	0.043*	
C13	0.7400 (4)	0.8609 (3)	0.44891 (14)	0.0491 (5)	
H13A	0.8105	0.9297	0.3995	0.074*	
H13B	0.7934	0.9171	0.5149	0.074*	
H13C	0.5815	0.8625	0.4447	0.074*	
O1	0.70435 (19)	0.58589 (15)	0.33028 (8)	0.0290 (3)	
O2	0.6007 (2)	0.31046 (17)	0.38231 (9)	0.0374 (3)	
C1	0.3008 (2)	0.27029 (19)	0.19204 (11)	0.0204 (3)	
C2	0.5237 (2)	0.35132 (19)	0.21333 (11)	0.0203 (3)	
C3	0.6587 (2)	0.36577 (19)	0.13282 (11)	0.0209 (3)	
H3	0.804	0.4231	0.1429	0.025*	
C4	0.5801 (2)	0.29487 (19)	0.03486 (11)	0.0193 (3)	
C5	0.7131 (2)	0.29932 (19)	−0.05125 (11)	0.0216 (3)	
H5	0.8595	0.3552	−0.0441	0.026*	
C6	0.6313 (2)	0.22322 (19)	−0.14494 (11)	0.0217 (3)	
C7	0.4081 (3)	0.1369 (2)	−0.15399 (11)	0.0242 (3)	
H7	0.3517	0.083	−0.2172	0.029*	
C8	0.2740 (2)	0.1305 (2)	−0.07286 (11)	0.0231 (3)	
H8	0.1283	0.0733	−0.0812	0.028*	
C9	0.3560 (2)	0.21049 (19)	0.02358 (11)	0.0198 (3)	
Cl1	0.11327 (6)	0.26599 (5)	0.29105 (3)	0.02749 (13)	
N1	0.2174 (2)	0.20436 (16)	0.10369 (9)	0.0218 (3)	

### Atomic displacement parameters (Å^2^)

**Table:**

	*U*^11^	*U*^22^	*U*^33^	*U*^12^	*U*^13^	*U*^23^
C10	0.0187 (7)	0.0291 (8)	0.0229 (8)	0.0065 (6)	0.0032 (6)	0.0034 (6)
C11	0.0327 (9)	0.0288 (8)	0.0235 (8)	0.0071 (7)	0.0035 (7)	0.0039 (6)
C12	0.0407 (10)	0.0442 (10)	0.0191 (8)	0.0002 (8)	−0.0072 (7)	−0.0014 (7)
C13	0.0723 (14)	0.0412 (11)	0.0298 (10)	0.0039 (10)	−0.0100 (9)	−0.0065 (8)
O1	0.0382 (6)	0.0265 (6)	0.0204 (5)	−0.0006 (5)	−0.0049 (5)	0.0009 (4)
O2	0.0440 (7)	0.0414 (7)	0.0278 (6)	−0.0020 (5)	−0.0028 (5)	0.0144 (5)
C1	0.0213 (7)	0.0180 (7)	0.0237 (8)	0.0053 (6)	0.0043 (6)	0.0061 (6)
C2	0.0212 (7)	0.0177 (7)	0.0231 (7)	0.0056 (6)	0.0015 (6)	0.0040 (6)
C3	0.0185 (7)	0.0188 (7)	0.0253 (8)	0.0014 (6)	−0.0005 (6)	0.0034 (6)
C4	0.0202 (7)	0.0144 (7)	0.0240 (7)	0.0040 (5)	0.0008 (6)	0.0039 (6)
C5	0.0190 (7)	0.0195 (7)	0.0270 (8)	0.0029 (6)	0.0014 (6)	0.0052 (6)
C6	0.0266 (8)	0.0169 (7)	0.0235 (7)	0.0070 (6)	0.0022 (6)	0.0058 (6)
C7	0.0306 (8)	0.0202 (7)	0.0218 (8)	0.0044 (6)	−0.0045 (6)	0.0023 (6)
C8	0.0217 (7)	0.0193 (7)	0.0279 (8)	0.0002 (6)	−0.0031 (6)	0.0043 (6)
C9	0.0209 (7)	0.0155 (7)	0.0241 (7)	0.0043 (5)	0.0005 (6)	0.0053 (6)
Cl1	0.0230 (2)	0.0336 (2)	0.0276 (2)	0.00604 (15)	0.00824 (15)	0.00777 (16)
N1	0.0200 (6)	0.0194 (6)	0.0267 (7)	0.0027 (5)	0.0020 (5)	0.0052 (5)

### Geometric parameters (Å, º)

**Table:**

C10—O2	1.1971 (18)	C1—C2	1.419 (2)
C10—O1	1.3308 (18)	C1—Cl1	1.7501 (14)
C10—C2	1.494 (2)	C2—C3	1.365 (2)
C11—C6	1.501 (2)	C3—C4	1.404 (2)
C11—H11A	0.96	C3—H3	0.93
C11—H11B	0.96	C4—C5	1.411 (2)
C11—H11C	0.96	C4—C9	1.421 (2)
C12—O1	1.4588 (19)	C5—C6	1.368 (2)
C12—C13	1.475 (3)	C5—H5	0.93
C12—H12A	0.97	C6—C7	1.420 (2)
C12—H12B	0.97	C7—C8	1.362 (2)
C13—H13A	0.96	C7—H7	0.93
C13—H13B	0.96	C8—C9	1.407 (2)
C13—H13C	0.96	C8—H8	0.93
C1—N1	1.2935 (19)	C9—N1	1.3673 (19)
			
O2—C10—O1	125.26 (14)	C3—C2—C1	116.71 (13)
O2—C10—C2	124.26 (14)	C3—C2—C10	121.18 (13)
O1—C10—C2	110.47 (12)	C1—C2—C10	122.05 (13)
C6—C11—H11A	109.5	C2—C3—C4	120.61 (13)
C6—C11—H11B	109.5	C2—C3—H3	119.7
H11A—C11—H11B	109.5	C4—C3—H3	119.7
C6—C11—H11C	109.5	C3—C4—C5	123.51 (13)
H11A—C11—H11C	109.5	C3—C4—C9	117.37 (13)
H11B—C11—H11C	109.5	C5—C4—C9	119.10 (13)
O1—C12—C13	107.55 (14)	C6—C5—C4	121.45 (13)
O1—C12—H12A	110.2	C6—C5—H5	119.3
C13—C12—H12A	110.2	C4—C5—H5	119.3
O1—C12—H12B	110.2	C5—C6—C7	118.37 (13)
C13—C12—H12B	110.2	C5—C6—C11	121.79 (13)
H12A—C12—H12B	108.5	C7—C6—C11	119.84 (13)
C12—C13—H13A	109.5	C8—C7—C6	121.99 (14)
C12—C13—H13B	109.5	C8—C7—H7	119
H13A—C13—H13B	109.5	C6—C7—H7	119
C12—C13—H13C	109.5	C7—C8—C9	119.98 (14)
H13A—C13—H13C	109.5	C7—C8—H8	120
H13B—C13—H13C	109.5	C9—C8—H8	120
C10—O1—C12	117.45 (12)	N1—C9—C8	118.90 (13)
N1—C1—C2	125.71 (13)	N1—C9—C4	122.00 (13)
N1—C1—Cl1	115.40 (11)	C8—C9—C4	119.10 (13)
C2—C1—Cl1	118.82 (11)	C1—N1—C9	117.48 (12)
			
O2—C10—O1—C12	−2.8 (2)	C9—C4—C5—C6	−0.4 (2)
C2—C10—O1—C12	178.41 (12)	C4—C5—C6—C7	−0.6 (2)
C13—C12—O1—C10	−140.62 (16)	C4—C5—C6—C11	−179.88 (13)
N1—C1—C2—C3	2.2 (2)	C5—C6—C7—C8	1.0 (2)
Cl1—C1—C2—C3	−174.62 (10)	C11—C6—C7—C8	−179.76 (13)
N1—C1—C2—C10	−174.93 (13)	C6—C7—C8—C9	−0.2 (2)
Cl1—C1—C2—C10	8.23 (18)	C7—C8—C9—N1	179.20 (12)
O2—C10—C2—C3	−123.43 (17)	C7—C8—C9—C4	−0.9 (2)
O1—C10—C2—C3	55.34 (18)	C3—C4—C9—N1	2.7 (2)
O2—C10—C2—C1	53.6 (2)	C5—C4—C9—N1	−178.89 (12)
O1—C10—C2—C1	−127.63 (14)	C3—C4—C9—C8	−177.25 (12)
C1—C2—C3—C4	−2.9 (2)	C5—C4—C9—C8	1.2 (2)
C10—C2—C3—C4	174.25 (12)	C2—C1—N1—C9	1.0 (2)
C2—C3—C4—C5	−177.71 (13)	Cl1—C1—N1—C9	177.90 (9)
C2—C3—C4—C9	0.7 (2)	C8—C9—N1—C1	176.48 (12)
C3—C4—C5—C6	177.92 (13)	C4—C9—N1—C1	−3.4 (2)
