# Supplementary figures and images for: Crystal structure of ethyl 2-chloro-6-methyl­quinoline-3-carboxyl­ate
Source: Acta Crystallogr Sect E Struct Rep Online. 2014 Aug 1;70(Pt 9):o941. doi: 10.1107/S1600536814016900 (PMC4186161; doi:10.1107/S1600536814016900)

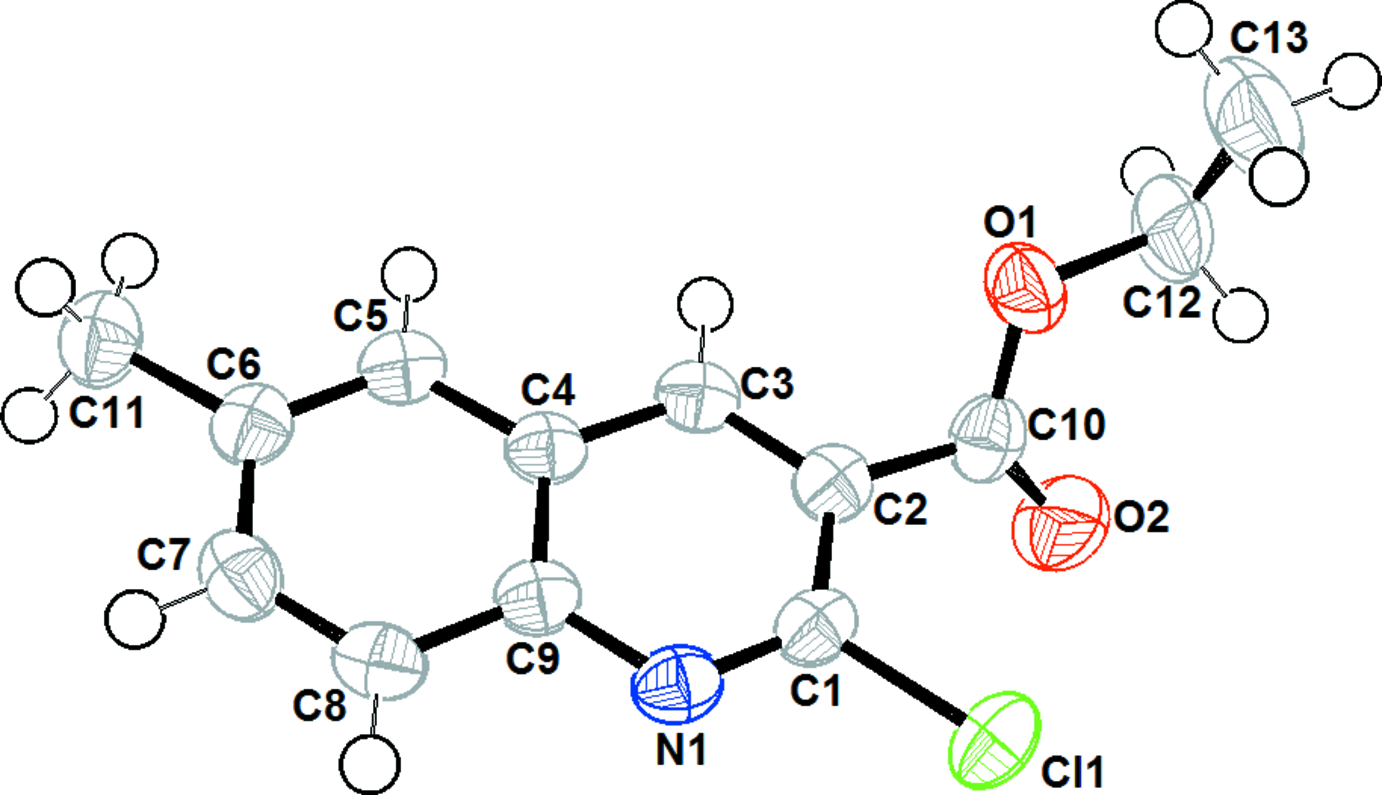

Supplement: Supplementary file 4 [file e-70-0o941-fig1.tif]

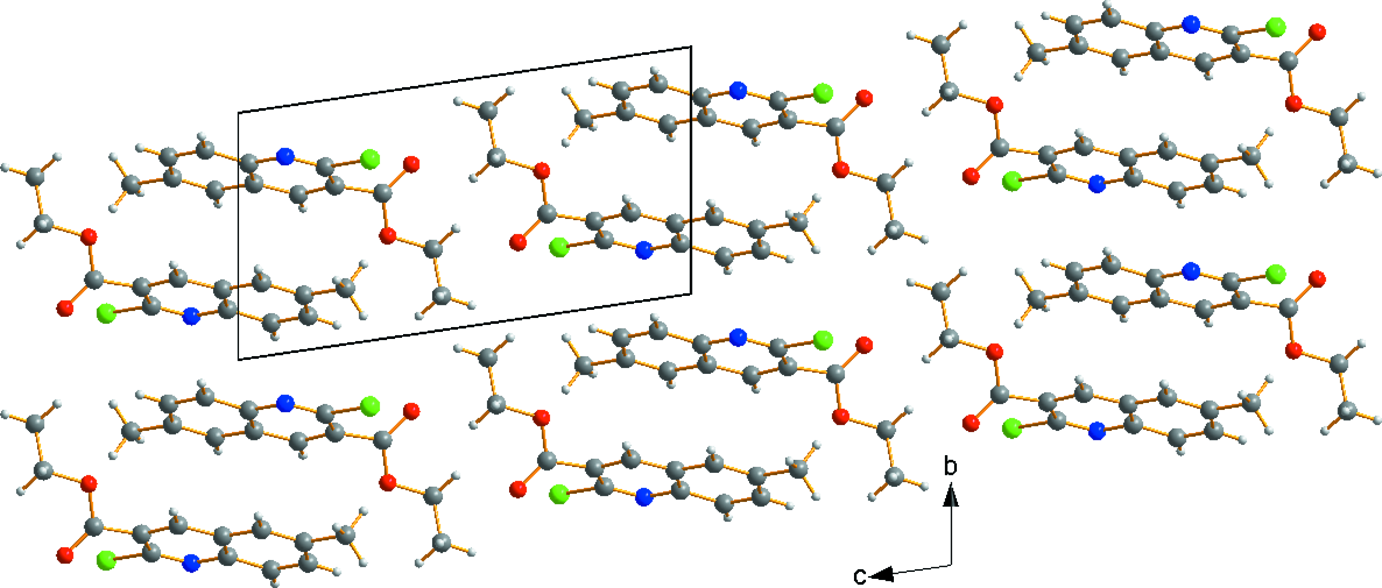

Supplement: Supplementary file 5 [file e-70-0o941-fig2.tif]
